# Application of Membrane Technology to Obtain Bioactive Products from Orange Peel Extract

**DOI:** 10.3390/foods14244202

**Published:** 2025-12-07

**Authors:** Asunción M. Hidalgo, José Antonio Macario Legaz, Jorge Saura-Martínez, Luis Tortosa-Díaz, Rubén López-Nicolás, Fulgencio Marín-Iniesta

**Affiliations:** 1 Department of Chemical Engineering, Regional Campus of International Excellence “Campus Mare Nostrum”, University of Murcia, 30100 Murcia, Spain; joseantonio.macario@um.es; 2 Group of Research Food Biotechnology-BTA, Department of Food Science, Nutrition and Bromatology, Regional Campus of International Excellence “Campus Mare Nostrum”, University of Murcia, 30100 Murcia, Spain; j.sauramartinez@um.es (J.S.-M.); luis.tortosad1@um.es (L.T.-D.); fmarin@um.es (F.M.-I.); 3 Biomedical Research Institute of Murcia (IMIB-Arrixaca), University of Murcia, 30003 Murcia, Spain; rubenln@um.es

**Keywords:** citrus waste, by-products, ultrafiltration membranes, nanofiltration

## Abstract

Orange peel is suitable for reuse due to the quantity and variety of bioactive compounds it contains, such as pectins, sugars and hesperidin. This study designed a scheme for reusing orange peel extract (OPE) using membrane technologies. Initially, a 100 kDa ceramic membrane was used to separate the pectins and hesperidine from acids and sugars and obtain a clarified product. In the subsequent stage, two ultrafiltration membranes of 25 and 5 kDa were tested, improving the results in terms of product transmittance and obtaining permeates whose physical–chemical parameters are compatible with those established by the European Fruit Juice Association. These membranes did not achieve complete separation of monosaccharide sugars from disaccharides. Finally, a 200 Da nanofiltration membrane was used, which completely reduced the sucrose and pectin content, concentrating glucose and fructose by 40%, values higher than those obtained with the GR90PP membrane. In addition, calcium and magnesium ions were completely rejected. Color changes in the permeate and concentrate streams could be appreciated due to the high concentration produced when working in batches. The nanofiltration (NF) process obtained lower yields (approximately 30%) compared to ultrafiltration (approximately 85%).

## 1. Introduction

The global production of oranges on 22 January 2021 was approximately over 48 million tons, with Brazil, China, the European Union, Mexico and the United States being the main producers. In several countries, oranges are mainly used for extracting juice, representing 1.7 million tons of their production until 22 January 2021 [1]. The global production of oranges in Spain in 2023 was approximately 2,715,000 tons [2]. Some important compounds present in orange fruit are folic acid, thiamine, riboflavin, niacin, pantothenic acid, B6, potassium, phosphorus, calcium, iron, magnesium, sodium, ascorbic acid, amino acids, flavonoids and phenolic compounds [3]. After its use, more than half of the fruit remains as a by-product. The orange peel represents the highest discarded waste [4,5], which is an important source of soluble sugar, pectin, ascorbic acid, fiber and phenolic compounds [6,7,8]. Seeds are another most discarded waste, which contains an important quantity of oil, protein, potassium, sodium, calcium, phosphorus, iron, ascorbic acid, oxalate, alkaloids, fatty acids, phytosterols, tocopherols and fiber [9,10,11,12]. Orange pomace is yet another waste with a large number of bio-functional compounds; however, its use requires special attention because its large amount of moisture makes it the most perishable waste due to its high susceptibility to microbial spoilage [13]. The proximate composition of orange by-products (including peel, seed, albedo and flavedo) includes ~60–70 of dietary fiber, 5.9–8.9 protein, 1.8–4.5 g/100 g dry matter of lipids and 534 mg GAE/100 g of dry matter of phenolic compounds [14,15,16]. These by-products can be revalorized as a natural source of vitamins, pectin, polyphenols, or essential oils with important health effects [17].

Emerging technologies, or technological processes, such as membrane technology, have been studied to find food applications for these by-products, given that they contain compounds with high added value, such as essential oils, antioxidants, pectins, etc., thus maximizing their economic value. [17,18]. Once they have been characterized and identified, they can be the starting point for the formulation and preparation of new bioactive products [3,19,20]. The recovery of the different fractions not only improves profitability but also helps to reduce environmental pollution by minimizing waste, as these actions are based on the maximal use of citrus fruits. [3,19,20]. Generally, for every ton of oranges, 553 kg of juice or 100 kg of orange concentrate at 65 Brix degrees is obtained. These values vary depending on the variety, weather conditions and condition of the fruit. Overall, 41.3% of the total weight is waste generated after juicing. This waste consists of peels, pulps and seeds with some juice impregnated in them. Due to its high content of organic matter, water and sugars, it is easily fermentable and represents a waste management problem in the industry [21]. The waste generated in the citrus industry has a high energy value, given its high concentration of carbohydrates. The most common use is for animal feed or fertilizer. This use is not entirely straightforward, as young animals are more reluctant and generally need to be accustomed to regular consumption, which is not possible in the summer months. In addition, transport is conditioned by the distance between farms and citrus companies and the weight of the water they contain. Another disadvantage is the corrosive power of the acids present, which can damage metals and cement. Other ways of adding value are limited by the high cost of drying, which makes their development and investment impossible [22]. Membrane technology is emerging as a replacement for traditional juice clarification and concentration processes, as they require less energy, reduce operating costs and operate at room temperature. Other advantages of these membrane processes over traditional methods are that they allow the product to be processed at lower temperatures (improving thermal stability), increase aroma retention and require less expensive equipment. [23]. The concentration of citrus juices not only provides microbiological stability but also allows for savings in the packaging and distribution of the finished product due to the reduction in weight and volume [24]. The potential advantages of membrane filtration (MF) and ultrafiltration (UF) processes over conventional filtration processes are obvious, given that they produce a higher quality product with low energy consumption [25].

Various authors have found that microfiltration and ultrafiltration treatments applied to citrus juice could produce a clarified juice in which suspended solids had been removed and most of the soluble solids and acids had been retained [26,27,28,29,30,31,32,33,34].

The main problem with using membrane processes in the clarification and concentration of citrus juices is membrane fouling. Membrane fouling manifests itself as a decrease in flow during operation, thus reducing the permeability of the membrane. The degree of fouling determines the frequency of cleaning, the membrane’s lifespan, the area required and, consequently, the design and operating costs of membrane plants [24]. Recently, the development of treatment methods for waste from citrus fruit processing has focused on separating functional macro- and micro-molecules with the aim of obtaining added value from these by-products. Membrane technologies are emerging as a tool to improve production “stock” and are the most suitable method for treating this waste [35,36]. Citrus flavonoids have been investigated for biological activities, including anti-inflammatory, anti-carcinogenic and anti-tumor, all of which have been demonstrated. Most citrus species accumulate substantial amounts of flavonoids during their development. All flavonoids described in citrus fruits can be classified into the following groups: flavanones, flavones and flavonols. Naringin (naringenin 7-O-neohesperidoside), a flavanone glycoside, is distinctly dominant in grapefruit (*C. paradisi*) and is responsible for the bitter taste of grapefruit juice. Narirutin (naringenin-7-O-rutioside) is also present in grapefruit, but in smaller proportions [37,38]. Table 1 shows the main compounds recovered as by-products of the citrus juice industry using conventional membrane processes. This table shows the separation process efficiency, membrane type, molecular cut-off size, configuration and the different bioactive compounds obtained for the different juices studied.

There are numerous studies that use microfiltration and nanofiltration to recover and concentrate these compounds of interest [41,42,43,44,45].

The aim of this study is to seek technological solutions aimed at making maximum use of the different fractions generated in orange fruit processing. The methodology followed involves the identification of each of the different fractions that are susceptible to valorization. Clarification processes involving membranes with different molecular cut-off sizes have been used as a means of recovering by-products and obtaining functional ingredients as hesperidin of pectins, which can be used as raw materials in beverages and formulations.

Orange peel pulp, which is a waste product of orange juice accounting for around 9% of the weight [46], causes economic and environmental problems due to its easy fermentation [47]. Clarified concentrated extracts from fruit juice have a high or low sugar content and extracts rich in antioxidants. Conventional clarification processes aim to remove insoluble solids and destroy pectic substances by degrading pectin and starch with specific enzymes and flocculating cloudiness with clarifying agents (bentonite, gelatin and/or silicasol) [48], being pectins the main cause of the turbidity present [49]. Membrane filtration ensures excellent permeate quality with increased clarity and, consequently, significant color removal [50]. In addition to the pectins, fibers and sugars mentioned above, another compound retained by successive filtrations is hesperidin. This compound is found in various citrus varieties. It is of interest for its cardioprotective, anti-inflammatory, neuroprotective and antioxidant properties [51,52]. All these compounds are of great interest for their nutritional and technological properties as ingredients that can replace additives in the food industry [53]. Taking advantage of the fractions retained during the filtration process, where these compounds are found, opens the possibility of new research into the revaluation of by-products, contributing to a reduction in waste following the circular economy model [54].

## 2. Materials and Methods

### 2.1. Raw Materials

The by-product used in this research is the orange peel extract (OPE). The OPE is composed of the fine pulp and the rest of the albedo, separated by filtration right after the fruit juicing. This mix is then processed with a pectinase enzymatic treatment and then concentrated to the corresponding Brix degrees. After all this procedure, you can obtain what is known in the industry as OPE. The most common values are from 50 to 65 Brix for orange juice.

Orange fruit juicing waste was obtained thanks to the AMC Global company (AMC Global, Murcia, Spain).

### 2.2. Reactives

- NaOH 0.1 M (Sigma-Aldrich; Berlin, Germany)
- Iodine 0.05 M (Sigma-Aldrich; Berlin, Germany)
- pH standards (4.01; 7.00; 9.21) (Sigma-Aldrich; Berlin, Germany)
- Maltose, Sucrose, D(-)-Fructose, D(+)-Glucose and D-Sorbitol standards (Sigma-Aldrich; Berlin, Germany)
- Starch/phenolphthalein dissolution (1% volume) (Sigma-Aldrich; Berlin, Germany)
- Potassium/sodium calibration dissolutions
- 2-Propanol technical grade (Sigma-Aldrich; Berlin, Germany)
- Acetonitrile (Panreac; Barcelona, Spain)
- Water HPLC grade
- Formaldehyde 37–38% *w*/*w* stabilized with methanol (Sigma-Aldrich; Berlin, Germany)
- Hesperidine, eriocitrin and limonin standards (Sigma-Aldrich; Berlin, Germany)

### 2.3. Membranes

Table 2 shows the main characteristics of the membranes used in this work.

### 2.4. Equipment

- Tangential filtration plant (Gea Westfalia model F2013, GEA Group, Düsseldorf, Germany). The F2013 tangential filtration equipment is designed for filtering juices, pectins, vegetable broths and wastewater with a capacity of 300–500 Lh^−1^. The system consists of a single-stage centrifugal pump that drives the feed to the membrane module. The plant has a stainless-steel strainer to prevent the entry of particles, as well as pressure and temperature indicators (range 0–120 °C), an inductive flow meter (to monitor the material retained in recirculation) and a stainless-steel tank. The unit is basically operated manually.
- Triple System Model F1 membrane module (MMS AG Membrane Systems, Urdorf, Switzerland) [55]. The membrane module used to perform the tests was the Triple System Model F1, manufactured by MMS, which has a maximum operating pressure and temperature of 40 bar and 50 °C, respectively. The experimental unit has a feed tank with a capacity of 800 mL, into which the feed solution for each test is introduced. A pump drives the feed to the three flat membrane modules. The pressure required for the filtration process to take place is supplied to the system by nitrogen gas.
- Nanofiltration plant (tangential filtration plant like Gea Westfalia model F2013, equipped with nanofiltration membrane GEA Group, Düsseldorf, Germany).

In addition to the filtration equipment used, some other equipment has been used to carry out or support the tests and parameterisation of the currents obtained. This equipment is shown in Table 3.

### 2.5. Experimental Methods

- Clarification through ultrafiltration 100 kDa

Tests were carried out at 50-, 40- and 30-degrees Brix (by diluting the undiluted product with water), but a continuous permeate was not obtained. The first drops of permeate were obtained at a concentration of 30 Brix degrees. Therefore, the final tests were carried out at 25 degrees Brix.

This involved introducing the OPE into the feed tank and manually increasing the flow rate of the feed pump until the maximum tolerable pressure values for the installation and membrane were achieved. A solution of 25 degrees Brix was used.

Each test was carried out in duplicate and lasted a total of three hours. As there was no cooling system, the temperature increased from 30 °C to a maximum of 60 °C; this is the maximum temperature at which the product will not degrade in terms of its sensory properties, given that the membrane can withstand temperatures of up to 90 °C.

- Optimisation of the clarified orange peel extract (COPE) using UF membranes (5 and 25 kDa)
  ○ Experimental series for initial membrane characterisation. Initially, the feed tank is filled with distilled water to determine the membrane’s permeability to the solvent.
  ○ Experimental series to determine the behaviour of the membrane towards the feed from the orange peel extract clarification. For each of the membranes tested (GR60PP and GR90PP), four tests are carried out, varying the operating pressure from 7 to 9 bar.
  ○ Experimental series for the final characterisation of the membrane: this consists of the same tests as the first experimental series, except that these are carried out after the experiment with the orange peel extract (COPE) clarification solution has been completed.
- Separation of sugars by nanofiltration membrane

A nanofiltration test was conducted using 155 kg of clarified product from the OPE permeate. For this purpose, a polyester-based polymer membrane was used in an 8-inch module with a molecular cut-off size of 200 Da. This membrane has a surface area of 2.5 m^2^. Initially, the conditions were modified during the first stage of the process until permeation of the product began. From this point onwards, samples were taken every 15 min from the permeate and reject streams. The initial product temperature was 6.5 °C, and the product was recirculated for conditioning. This involved heating the product with steam to a temperature of 16.5 °C, resulting in a decrease in Brix degrees to 19.2. The steam was then stopped.

### 2.6. Analytical Methods

The analytical methods used in the physicochemical characterization of the different samples are compiled in Table 4.

## 3. Results and Discussion

### 3.1. OPE Characterization

**Figure 1:**
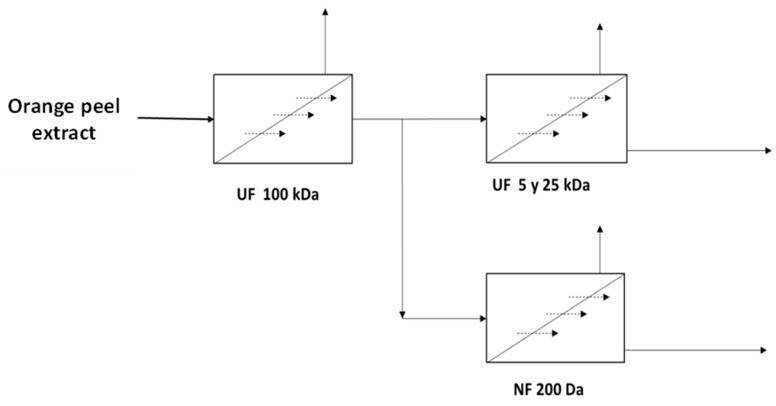
Block diagram of the stages carried out in the membrane study.

This work has been carried out according to the diagram in Figure 1, which shows the different stages involved in separating the different components. Diluted OPE was passed through an ultrafiltration membrane (100 kDa) to obtain a clarified product. Subsequently, the permeate stream was optimized by passing it through ultrafiltration membranes with a smaller cut-off size than the previous one (5 and 25 kDa) to obtain a more clarified permeate, given that consumers are increasingly demanding more restrictive specifications. Additionally, the product of the first filtration with the 100 kDa membrane was introduced into a nanofiltration stage, which seeks to separate the different sugars (mono from disaccharides) present in the by-product, thus obtaining natural sugars from the fruit.

### 3.2. Clarification Through Ultrafiltration 100 kDa

The main objective of this stage was to clarify the product. This process can increase short-term profitability as it allows for wider use, mainly because OPE usually has a strong bitter taste and toasted aromas. This stage makes it possible to increase the production of this by-product, thereby increasing the profitability of the process, given that it is currently not possible to use all the by-products when the plant is operating at 100% capacity, and in these cases, part of the peel is discarded. This process could enable the subsequent use or reuse of all skins.

According to the literature consulted, using raw material from orange and bergamot peel, authors Ruby-Figueroa et al. [39] and Conidi et al. [44] chose to clarify the product using 100 kDa ultrafiltration membranes. Similarly, in this study, the same molecular cut-off size was selected. Table S1 shows the experimental conditions. The average permeate flow rate obtained throughout the test was 53 Lh^−1^.

The results obtained in the tests are shown in Table 5, which shows the main parameters of the feed, permeate and concentrate streams. Before entering the first membrane, OPE had to be diluted in a 1:1(volume/volume, (*v*/*v*)) proportion with distilled water to be able to pass through the membrane.

In Table 5, we can see the main parameters of the OPE used as a raw material in the experiments.

Table 5 shows that the percentage of acidity, formaldehyde index, potassium and pH remained virtually unchanged, while pulp and transmittance varied significantly due to the clarification process, which retained the pulp content and most of the pectins that are the main causes of turbidity, as indicated by Dey and Banerjee [49].

The aim of the test was to obtain the maximum Brix degrees that can be introduced into the filtration equipment operating at 100% efficiency to save energy costs in the subsequent concentration stage. In addition to concentration costs, there are also savings in logistics costs due to the volume of raw material transported and in storage tanks due to the lower volume of work. Table 6 shows the variation in % transmittance throughout the process.

Table 6 shows a decrease in the percentage of transmittance as the product permeates. However, throughout the test, it remained above 95%, a value at which clarification is considered to have occurred correctly. According to Jegatheesan et al. [50], membrane filtration can achieve excellent quality permeate streams and almost total removal of turbidity and color.

In view of the results in Table 5 and Table 6, it was determined that with approximately 25 Brix degrees in the input sample, the membrane can clarify the product throughout the test with transmittance values in accordance with market specifications, which are established at a transmittance percentage greater than 95%.

Table 7 shows the sugars present in the permeate and feed compared to the values set by the European Fruit Juice Association (AIJN) for a standard orange juice analyzed at 11.2 Brix degrees. AIJN has been the representative association for the fruit juice industry in the EU since 1962 and produces reference guides for each type of fruit, including the usual values for the main parameters related to composition, environmental and hygiene requirements. Standard orange juice is analyzed at 11.2 Brix degrees.

The increase in monosaccharides during clarification (glucose and fructose) is closely related to the decrease in the sugar-free extract, since part of the composition of this extract was made up of the majority of pectins, pulp and flavonoids, among others, which were rejected in the membrane, while mono- and disaccharides passed through the membrane.

In both cases, sucrose and the glucose/fructose ratio were unbalanced. This is because the composition of sugars present in the peel was not entirely like that of the juice, as well as because of the pectinase enzymatic extraction itself, as pointed out by Maktouf et al. [32]. Finally, Table 8 shows the specific parameters analyzed.

**Figure 2:**
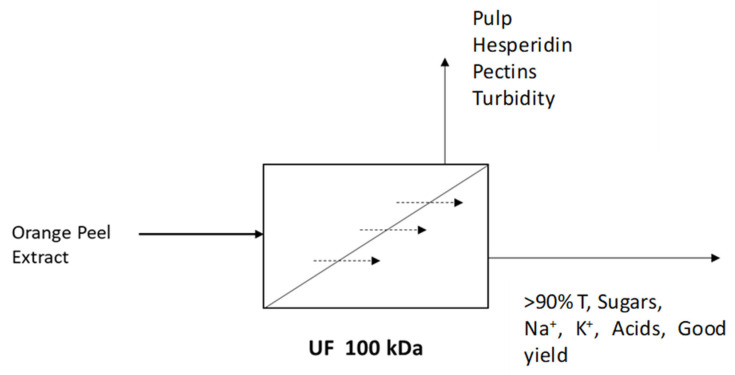
Ultrafiltration process streams with the most representative products.

Among the values represented in the table above, the reduction in hesperidin and total and water-soluble pectins stands out. AIJN, in its guide for oranges, already indicates the reduction in hesperidin due to the clarification process, since the molecular weight cut-off of the membrane does not allow this flavonoid to pass through, remaining in the reject stream. This recoverable stream may be useful in the future for the extraction of this and other high-value-added compounds. The low proportion of free sugars in this stream may also be useful for certain food applications, given current trends toward sugar in foods. There is also a significant decrease in total and water-soluble pectins. The consequence of the reduction in pectins was described by Kashyap et al. [67] as a reduction in turbidity. Figure 2 describes the different streams of the ultrafiltration process with the most representative products present.

### 3.3. Optimisation of the Clarified Orange Peel Extract (COPE) Using UF Membranes (5 and 25 kDa)

The feed stream came from the product clarified by the ultrafiltration process using a membrane with a molecular cut-off size of 100 kDa.

The aim of this stage was to optimize the permeate stream obtained by the concentration of the sugars present. To this end, from the previous permeate, tests were carried out using two membranes of lower molecular cut-off size, 25 and 5 kDa, and of different chemical composition, one of polysulphone and the other of polyethersulphone, respectively. The overall objective of these tests was to study the possibility of separating sugars of larger molecular size (disaccharides) from those of smaller size (monosaccharides), as well as the possibility of concentrating the sugars in the rejection stream.

In addition, these trials sought to optimize the permeate from clarification using a 100 kDa MWCO ultrafiltration membrane in order to improve its organoleptic profile. It should be noted that, by using an ultrafiltration process compared to a nanofiltration process, energy consumption is considerably reduced as it operates at lower pressures.

As a preliminary step, the initial characterization of the membranes was carried out by performing a solvent permeability study. To characterize the membranes, the permeability of each membrane to the solvent was calculated before and after the tests with the COPE.

Equation (1) expresses the flow of water through the membrane by means of a driving force, which is the pressure gradient:

(1) $$J_{w}=A_{w}\;\times \left(\Delta P-\Delta \Pi \right)$$

where

*A_w_*: water permeability coefficient (hm^−1^).

*J_w_*: water permeate flux (m^3^m^2^·h^−1^).

*∆P*: hydraulic pressure gradient (bar).

*∆Π*: osmotic pressure gradient (bar).

#### 3.3.1. Determination of Permeability by Testing Distilled Water

Experimentally, it was observed that there was a linear relationship between the solvent flux and the pressure gradient across the membrane when testing with water. In this case, there are no salts or organic solutes in the feed. When the solute concentrations are low, the osmotic pressure gradient is very low and can be neglected compared to the hydraulic pressure gradient. Thus, Equation (1) will be reduced to (2):

(2) $$J_{w}=\;A_{w}\;x\;∆P$$

By representing the permeate flux (*J_w_*, in m^3^ (m^2^·h)^−1^) versus the hydraulic pressure (bar) and making a linear regression adjustment, we obtain a straight line whose slope is equal to the permeability of the membrane to water (*A_w_*, in hm^−1^). It must be considered, when making the calculations, that the useful area of the membrane coupled to the module is 8.48 × 10^−3^ m^2^.

**Figure 3:**
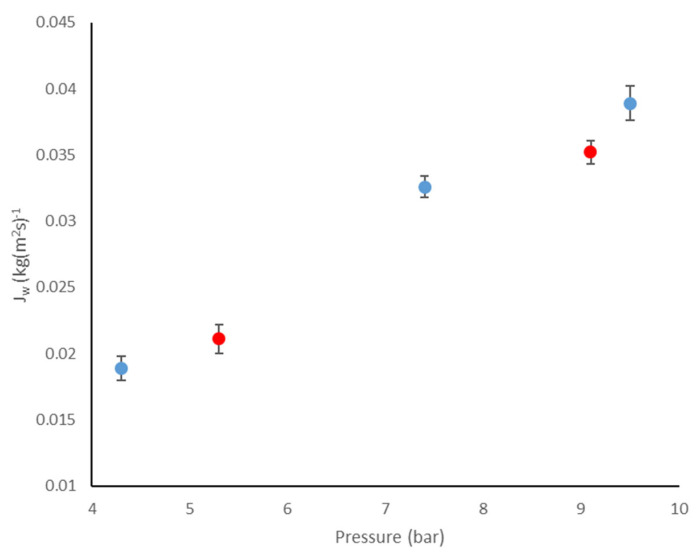
Permeability test for GR60PP membrane before (●) and after (●) passing the COPE. Mean (*n* = 2) ± SD.

Several tests were carried out with water to experimentally determine the permeability (*A_w_*) of the GR60PP and GR90PP membranes. For this purpose, the operating pressure varied between 4 and 10 bar and the temperature was kept constant at 25 ± 5 °C. With the experimental data obtained, the graphical representation and the relevant linear adjustment were made, thus obtaining the permeabilities of each membrane. In addition, a comparative study was carried out between the experimental permeability data obtained for each membrane, before and after the COPE step. Figure 3 shows the results of the permeability test for the GR60PP membrane.

The two membranes used (GR60PP and GR90PP) were compared with the literature. Table 9 shows the permeability values of the membranes tested and the literature consulted for the distilled water tests.

Table 9 shows that the values of the permeability coefficients obtained experimentally and those consulted in the literature are of the same order. Sánchez-Moya et al. [68] used GR60PP and GR90PP membranes for the separation of lactose and whey protein, obtaining values of the permeability coefficient (*A_w_*) in sm^−1^ of the same order of magnitude as those obtained experimentally for these membranes. In another study, similar values were obtained with the GR60PP membrane by Murcia et al. [69].

The GR60PP (polysulfone) membrane is composed of sequential aromatic and aliphatic units, which give the polymer its hydrophobic profile by repelling water and hydrophilic compounds. Occasionally, this structure has a hydrophilic character due to the formation of hydrogen bonds through the connection between oxygen molecules (aryl-O-alkyl) and sulphur dioxide (aryl-SO_2_-alkyl). On the other hand, the structure of polyethersulfone (GR90PP) is similar but less hydrophobic due to the presence of a greater number of sulphur dioxide molecules [70]. This is because the oxygen atoms present in the SO_2_ molecule can bind to water. In addition, membranes with a polyethersulfone chemical composition are resistant to proteins and polysaccharides [71,72].

#### 3.3.2. Characterization of the Permeate and Concentrate Streams

Once the filtration tests were carried out, the results of the analysis of the feed and permeate streams for each membrane tested are shown in Table 10.

Table 10 shows that the Brix degrees decreased slightly due to the elimination of Brix-contributing compounds, but the pH, acidity percentage, formaldehyde index, potassium and ascorbic acid remained unchanged. Castro et al. [73] observed that the antioxidant capacity after membrane filtration remained unchanged and even higher than traditional filtration by diatomaceous earth. These results are in line with the data obtained in the trials, where ascorbic acid remained at approximately the same levels after passing through the membrane.

With this ultrafiltration process, turbidity and viscosity were reduced, as occurred in the study by Maktouf et al. [32]. A more transparent product with a more neutral appearance and flavor was obtained, reducing the perception of bitterness, being the membrane with the smallest pore size (GR90PP), the one that obtained higher transmittance percentage values.

This way, a product with as little flavor or color as possible could be applied in different references and not limit its use, while maintaining the minerals and acids present. Luo et al. [74] justified that a passage through tight ultrafiltration after loose ultrafiltration was able to reduce pigments and thus obtain juice with a low coloring. The terms “loose and tight” correspond to a larger and a smaller pore size membrane.

Table 11 below shows the sugar analysis of the different feed, permeate and reject streams.

The increase in the Brix degrees presented by the rejection streams compared to the permeate streams in both membranes is due to the fact that the system works in batches, i.e., a quantity of product is introduced into the membrane feed tank and permeate is obtained, while the rejection is returned to the tank, producing a concentration of the soluble solids present.

In order to discuss the results of the sugar concentration, the sugar concentration values must be referred to 11.2 Brix degrees, thus obtaining Table 12, where a comparative column is included with the usual ranges of orange juice according to AIJN.

In the above table, values of sucrose and glucose/fructose ratio are altered due to the composition of the sugars present in the orange peel (flavedo and albedo), which is not entirely similar to orange juice, and which is compared in the last column. Similar values were obtained when clarifying the OPE and in the studies carried out by Maktouf et al. [32].

On the other hand, the differences in the value of the sugar-free extract in the rejection streams versus the permeate streams in both membranes can be seen, as the sugars passed through the permeate stream while other compounds, such as pectins, flavonoids and part of the sucrose, remained in the rejection stream. The sucrose percentage and the sugar-free extract were obtained by Equation (3).

To determine how efficiently monosaccharides were separated from disaccharides, the percentage of rejection of the different sugars (a parameter related to the degree of concentration of the feed) was calculated using Equation (3), following the equation that Conidi et al. [76] used to study the membrane rejection of different phenolic compounds in pomegranate juice clarified by ultrafiltration and nanofiltration membranes. In addition, the percentage concentration was calculated using Equation (4). These values are shown in Table 13.

The formulas used were as follows:

(3) $$\%\;Rejection=\frac{C_{a}-C_{p}}{C_{a}}\times 100$$

(4) $$\%\;Concentrate=\frac{C_{r}}{C_{a}}\times 100$$

where

*C_a_*: Feed concentration (g L^−1^)

*C_p_*: Permeate concentration (g L^−1^)

*C_r_*: Reject concentration (g L^−1^)

In membrane-based juice clarification processes, negative rejection coefficients can be observed, particularly for small solutes such as sugars and organic acids. This is due to phenomena specific to complex matrices, such as concentration polarisation, the formation of a gel layer and the release of retained solutes. The results obtained indicate that the GR90PP membrane, with a smaller molecular cut-off size, was able to reject up to 15% of the disaccharides, while for the GR60PP membrane, the percentage composition of disaccharides remains practically unchanged. On the other hand, both membranes produced a concentration of around 14% of fructose in the permeate streams and from 4 to 7% for glucose. Therefore, if the separation of monosaccharide sugars from disaccharides to a certain extent is required, the best option would be the GR90PP membrane, which is capable of a higher degree of separation than the GR60PP membrane.

These results are in accordance with the bibliography. Ahmed et al. [77] investigated a combined membrane filtration approach for recovering high-quality water from streams of by-products from the beet sugar industry. The authors commented that the GR90PP membrane achieved a significant rejection of neutral sugars, with a rejection rate of 6.4% for sucrose (with a molecular weight of ~342 Da) and 12.0% for raffinose (with a molecular weight of ~504 Da), despite both being smaller than the nominal MWCO. According to the authors, the membrane may exhibit an effective MWCO of less than 5 kDa, or additional factors such as steric hindrance, solute shape and hydrophilic–hydrophobic interactions may influence transport.

#### 3.3.3. Fouling Study

One of the problems that membranes encounter during operation is related to fouling phenomena. To understand the fouling phenomena that membranes suffer during their use, a study was carried out in which the GR60 PP membrane was selected due to its chemical composition based on polysulfone, which is very common in the field of polymeric ultrafiltration and nanofiltration membranes.

To this end, the solvent permeability coefficient values were obtained after passing the COPE solution. The comparative study of the experimental data obtained at the beginning and end of the tests showed that the permeability coefficients are of the same order as those consulted in the literature [78].

On the other hand, Equations (5) and (6) were used to assess the degree of irreversible fouling (IF) and irreversible fouling rate of the membrane, respectively, as described by Warczok et al. [79] and Echavarría et al. [34] in their studies, where the permeate fluxes with distilled water before and after the membrane was used. Equation (6) was used to define the fouling index (FI) of the membrane, employed by Conidi et al. [76], where the permeability of the membrane was compared by the passage of distilled water before and after the passage of the juice. The results of the fouling index and irreversible fouling for each of the membranes used are presented in Table 14.

(5) $$IF=\frac{J_{wi}-J_{wf}}{J_{wi}}$$

(6) $$FI=\left(\frac{A_{wf}}{A_{wi}}\right)\times 100$$

where

*A_wi_*: initial water permeability coefficient (s m^−1^)

*A_wf_*: final water permeability coefficient (s m^−1^)

*J_wi_*: initial water permeate flux (kg (m^2^·s)^−1^)

*J_wf_*: final water permeate flux (kgm^2^·s)^−1^)

**Table 14:** Fouling index and irreversible fouling of the GR60PP and GR90PP membranes used.

Membranes	FI (%)	IF
GR60PP	94.92 ± 1.42	0.095 ± 0.001

The data obtained indicates that a value of 94.92% was reached for the fouling index (FI) in the GR60PP membrane, which is a very positive value. The fouling index indicates how much permeability drops after the product has passed through, but before the membrane is regenerated; values above 90% are acceptable. On the other hand, the degree of irreversible fouling (IF) for the GR60PP membrane was 0.095. This low value means that the membrane was not affected by the passage of the product. These excellent results obtained for both the fouling index and irreversible fouling are largely due to using raw material from a previous clarification. Other authors such as Conidi et al. [76] and Echavarría et al. [34] obtained lower data for the FI and higher data for the IF because they used juice without previous clarification. Because of the results obtained for the fouling index and irreversible fouling, it could be possible to lengthen the cycles between each cleaning in time, thereby increasing production.

This section may be divided by subheadings. It should provide a concise and precise description of the experimental results, their interpretation, as well as the experimental conclusions that can be drawn.

### 3.4. Separation of Sugars by Nanofiltration Membrane

Currently, in European Union countries, juice drinks are required to contain only naturally occurring sugars. Furthermore, with the new law taxing sugary and sweetened drinks, it is even more important to obtain natural sugars from fruit. That is why this study proposed separating naturally occurring sugars from orange and lemon peel extracts, which are by-products of the citrus industry.

As in the previous sections, membrane filtration was used, but in this case, a pore size that would allow the separation of sugars was sought. Therefore, nanofiltration membranes with a pore size of 200 Daltons were considered for the tests.

At the start of the test, the permeate flow rate was at its highest, decreasing as the experiment progressed. Conversely, the Brix degrees of the permeate and reject streams increased (Table S2 shows experimental results of the nanofiltration test).

This evolution in both streams could be due to various factors, such as the evolution of pressure and temperature during the test, as there was no cooling column available to keep these variables constant. The increase in temperature was studied by Álvarez-Quintana et al. [80], who found that it caused two effects: the first one is a decrease in viscosity, which facilitated the passage of product through the membrane; the second, which was related to the first, is a widening in pore size of the membrane. Similarly, Soltane et al. [81] studied how an increase in pressure can facilitate the passage of compounds into the permeate stream by modifying the structure.

The main sugars present, such as glucose, fructose and sucrose, were analyzed using high-performance liquid chromatography. In order to compare the concentrations of the different sugars, Table 15 expresses them in g kg^−1^ of dry matter (g kg^−1^ DM).

Table 15 shows the different concentrations of monosaccharides and disaccharides present in COPE and how they are distributed after the nanofiltration process. Luo et al. [74] found that nanofiltration membranes could separate monosaccharide sugars from disaccharides. Similarly, the table shows that no sucrose was detected in the permeate stream, with a membrane rejection coefficient of 100%, resulting in its total elimination, while around 40% of glucose and fructose are concentrated. Membrane rejection has been calculated using Equation (3). In contrast, sucrose was concentrated in the rejection stream, and the glucose and fructose slightly decreased. García-Martín [82] also found that passing musts and wines through nanofiltration reduced sugars by 30–60%, depending on the membrane used, operating conditions and the amount of sugars initially present.

It should be noted that nanofiltration can remove remaining pectins and reduce almost all divalent ions, such as calcium or magnesium, while monovalent ions, such as sodium or potassium, pass through the membrane. Furthermore, malic acid, with a molecular weight of 134.09 g moL^−1^, and ascorbic acid, with a molecular weight of 176.12 g moL^−1^, are able to pass through the membrane.

## 4. Conclusions

The COPE obtained through the 100 kDa ceramic membrane has a transmittance of over 95%. The membrane retains 85% of the pulp and pectin content, which are responsible for turbidity. The permeate complies with the usual physical–chemical parameters for juices and derivatives. In addition, there is an increase in monosaccharides in the clarified product (glucose and fructose), linked to the decrease in sugar-free extract, which in this case consists of pectins, pulp and flavonoids. The reduction in hesperidin is noteworthy.

These compounds are separated into different fractions throughout the filtration process. These fractions may have interesting uses as food ingredients that replace additives, opening up different avenues for future research.

When optimizing the UF membranes (MWCO 25 and 5 kDa) to treat the clarified stream from the previous stage, it was observed that permeate flows were high because the feed came from a previous clarification stage. In all cases, the transmittance of the product has increased to over 99%. In addition, the physical–chemical parameters of the permeates decreased slightly, although the pH, percentage of acidity, formaldehyde index, K^+^ and ascorbic acid remained unchanged. However, the monosaccharides were not completely separated from the disaccharides.

During the NF process using the 200 Da membrane, tests showed that the sucrose and pectin content was completely reduced, concentrating glucose and fructose to 40%, values higher than those obtained with the GR90PP membrane. On the other hand, Ca^+2^ and Mg^+2^ ions are completely rejected. The higher conductivity values in the permeates were due to the presence of K^+^ ions that passed through the membrane.

The color changes in the permeate and reject streams were due to the high Brix concentration that occurs when working in batches. It should be noted that the NF process had yielded 30% lower than those of UF.

## Figures and Tables

**Table 1:** The main compounds recovered as by-products of the citrus juice industry using conventional membrane processes. This table shows the separation process efficiency, membrane type, molecular cut-off size, configuration and the different bioactive compounds obtained for the different juices studied.

Compounds	Separation Efficiency	By-Product	Process	MWCO	Material	Configuration	References
Polyphenols	58.30%	Orange press liqueur	UF	100 kDa	Polysulfone	Hollow fiber	[39,40]
Anthocyanins and flavonoids	>90%	Orange press liqueur	NF	180 Da	Polyamide-polysulfone	Spiral module	[41]
	>80%		NF	300 Da	Thin-layerpolypiperazine amide		
	>80%		NF	400 Da	Polyethersulfone		
	>70%		NF	1000 Da	Polyethersulfone		
Anthocyanins and flavonoids	>65%	Orange press liqueur	NF	Rejection Na_2_SO_4_ > 25–50%	Polyethersulfone	Spiral module	[42]
Carotenes and flavonoids		Orange and clementine juice	MF + DF	0.2 µm	Ceramics	Tubular module	[43]
Polyphenols and flavonoids	91–97% flavonoids	Bergamot juice	UF + NF	100 kDa + 450 Da	Polysulfone + TiO_2_	Flat membrane Hollow fiber Tubular module	[44]
	43–62% polyph. 44%			100 kDa + 750 Da	Polysulfone + TiO_2_		

**Table 2:** Description of the membranes used in this work.

	Clarification Membrane	Optimization Membrane	Optimization Membrane	Sugar Separation Membrane
Supply company	Pall Corporation (Port Washington, NY, USA)	Alfa Laval Iberia S.A (Madrid, Spain)	Alfa Laval Iberia S.A (Madrid, Spain)	Alfa Laval Iberia S.A (Madrid, Spain)
Commercial name	Membralox	GR60PP	GR90PP	NF
Membrane type	Multichannel ceramic	Thin composite polypropylene layer	Thin composite polypropylene layer	Polymeric
Chemical composition	Zirconium	Polysulfone	Polyetersulfone	Polyester
Surface area (m^2^)	3	8.48 × 10^−3^	8.48 × 10^−3^	2.5
Maximum pressure (bar)	5	10	10	55
Maximum temperature (°C)	90	75	75	60
pH range	1–13	1–13	1–13	2–9
MWCO (Da)	100,000	25,000	5000	200

**Table 3:** Description of additional equipment used in this work.

Equipment	Model	Measure
Refractometer	Atago RX5000 α-BEV (Atago Co., Ltd., Tokyo, Japan)	Brix degrees
pH meter	Hach SensION (Hach Company, Loveland, CO, USA)	pH
Automatic calibrator	Methrom 916 Ti-Touch (Methrom AG, Herisam, Switzerland)	Acidity, formol index
Spectrophotometer	Thermo Scientific Genesys 10 (ThermoFisher Scientific, Waltham, MA, USA)	Absorbance, transmittance
Flame photometer	Jenway pfp7 (AntyliaScientifica, Vernon Hills, IL, USA)	Sodium, potassium
Turbidimeter	Hach 2100AN (Hach Company, Loveland, CO, USA)	Turbidity NTU
Centrifuge	Hettich rotofix 3 (Andreas Hettich Gmbh, Tutlingen, Germany)	Pulp content
HPLC	Merck LaChrom (Merck KGaA, Darmstadt, Germany)	Sugars
HPLC	Agilent 1100 series (Agilent Technologies, Santa Clara, CA, USA)	Limonin, eriocitrine, hesperidine
Laboratory scale	Radwag FS 4500 (Radwag, Radom, Poland)	Mass
Precision scale	Sartorius CP/245 (Sartorius AG, Gotinga, Germany)	Mass
Scale	Omron BF-508 (Omron Corporate, Kyoto, Japan)	Mass
Freezer	Beko (Beko Elektronik Karman, Istambul, Turkey)	-
Refrigerator	Beko (Beko Elektronik Karman, Istambul, Turkey)	-
Dry column height gauge	-	Height
Laboratory pasteurizer	Inoxpaser (Inoxpaser, S.L., Murcia, Spain)	-

**Table 4:** List of the different methods used for each analytical determination.

Methods	Analytical Determinations
IFU nº 8. Determination of soluble solids (indirect method by refractometry) [56]	Brix degrees determination in juices
IFU nº 33. Determination of sodium, potassium, calcium and magnesium [57]	Sodium/potassium content determination
IFU nº 11. Determination of pH value [58]	pH determination
JBT. Cap IV. Nº 27. Ascorbic acid by iodine determination [59]	Vitamin C (ascorbic acid) determination
IFU nº 3. Tritable acidity and IFU nº 30. Determination of formol number [60,61]	Acid percentage and formol index determination
IFU nº 60. Determination of centrifugable pulp [62]	Pulp content in juices determination
Determination of sugars by HPLC [63]	Identification and quantification of sugars
IFU Recomendation nº 7. Turbidity measurements [58]	Turbidity test
IFU nº 80. Measurement of the color of clear and hazy juices [64]	Absorbance and transmittance measurement
JBT. Cap.IV, nº 30. Limonin by HPLC [65]	Limonin determination

**Table 5:** Main parameters of the different streams: raw material, feed, permeate and reject for OPE.

Parameters	Raw Material (OPE)	Feed Solution (Diluted OPE)	Permeate Stream	Concentrate Stream
Brix	49.47 ± 1.98	25.0 ± 1.1	22.0 ± 0.8	22.8 ± 0.9
Acidity (%ACA)	2.48 ± 0.08	1.19 ± 0.04	1.1 ± 0.1	1.16 ± 0.04
pH	3.51 ± 0.14	3.55 ± 0.14	3.60 ± 0.15	3.57 ± 0.15
Formaldehyde index (mL100 mL^−1^)	16.10 ± 0.80	15.75 ± 0.79	15.96 ± 0.79	15.88 ± 0.79
* Pulp (%vv^−1^)	0.6 ± 0.1	0.6 ± 0.1	0	1.1 ± 0.2
* Potassium (mg L^−1^)	1337 ± 20	1312 ± 19	1324 ± 20	1351 ± 20
* Transmittance a 650 nm (%)	0.10 ± 0.01	0.20 ± 0.01	95.8 ± 1.9	0.10 ± 0.01
Color	Pale orange	Pale orange	Golden	Pale orange
Flavour	Orange peel	Orange peel	Bitter orange marmalade	Orange peel
Aroma	Citrus	Citrus	Citrus	Citrus
cleanliness/defects	OK	OK	OK	OK

(*) Tests carried out at 11.2 Brix degrees. Mean (*n* = 2) ± SD.

**Table 6:** Evolution of transmittance during the clarification process at 650 nm.

t (min)	%T_650_
Unclarified sample	0.2 ± 0.01
15	98.02 ± 1.96
60	98.13 ± 1.96
120	97.91 ± 1.96
205	97.95 ± 1.96
Final rejection	0.1 ± 0.01

Mean (*n* = 2) ± SD.

**Table 7:** Sugar content of different clarified products.

Sugars	Diluted OPE (g L^−1^)	Clarified OPE (g L^−1^)	Orange Juice (g L^−1^) [66]
Fructose 2%	24.42 ± 0.37	28.32 ± 0.42	20–27 *
Glucose	26.15 ± 0.39	31.56 ± 0.47	18–25 *
Sucrose	18.88 ± 0.28	20.30 ± 0.30	*25–55*
Maltose	0.00	0.00	*--*
Sorbitol	0.00	0.00	*--*
Isomaltose	0.00	0.00	--
% Sucrose	27.19 ± 0.41	25.30 ± 0.38	*≤55*
Glucose/fructose	1.07 ± 0.02	1.11 ± 0.02	*0.85–1.00*
Sugar-free extract	48.70 ± 0.73	37.80 ± 0.58	*24–40*

(*) Mediterranean orange juices may contain glucose and fructose levels up to 35 g L^−1^. Mean (*n* = 2) ± SD.

**Table 8:** Specific parameters of clarified OPE.

Parameters 2%	Diluted OPE (g L^−1^)	Clarified OPE (g L^−1^)	Orange Juice (g L^−1^): AIJN (Rev. June 2024)
Hesperidin (mg L^−1^)	1203 ± 18	78.84 ± 1.18	250–700
Limonin (mg L^−1^)	2.10 ± 0.03	1.50 ± 0.02	-
Eriocitrin (mg L^−1^)	0	0	-
Citric acid (g L^−1^)	10.60 ± 0.16	10.00 ± 0.15	6.3–17
D-Iso-citric acid (mg kg^−1^)	97.07 ± 1.45	78.91 ± 1.18	65–200
Citric/Iso-citric ratio	109.20 ± 1.64	126.7 ± 1.9	Max 130
Total pectins (expressed as monogalacturonic acid)	14,467 ± 217	1671 ± 25	-
Water-soluble pectins (mg kg^−1^)	9000 ± 135	1589 ± 24	200–500

Mean (*n* = 2) ± SD.

**Table 9:** Comparison of the water permeability coefficients of the different membranes (those obtained experimentally and those consulted in the bibliography).

Membranes	Experimental *A_w_* (sm^−1^)	Bibliography *A_w_* (sm^−1^)	
GR60PP	1.083 × 10^−8^	6.69 × 10^−8^	6,41 × 10^−8^
GR90PP	1.056 × 10^−8^	5.06 × 10^−8^	--
References	This work	[68]	[69]

**Table 10:** Main physicochemical parameters of the feed stream and the permeates obtained.

Parameters	Feed Stream (COPE)	GR60PP Permeate	GR90PP Permeate
Brix degrees	22.0 ± 0.8	19.5 ± 0.8	18.28 ± 0.70
Acidity (%ACA)	1.10 ± 0.04	1.00 ± 0.04	1.00 ± 0.04
pH	3.60 ± 0.14	3.63 ± 0.15	3.69 ± 0.15
Formaldehyde index (ml 100 mL^−1^)	15.96 ± 0.80	15.66 ± 0.78	15.84 ± 0.8
* Potassium (mg L^−1^)	1324 ± 20	1235 ± 18	1301 ± 19
* Ascorbic acid (mg100 mL^−1^)	70.4 ± 3.1	68.9 ± 2.8	71.1 ± 2.9
* Transmittance a 650 nm (%)	95.8 ± 1.9	97.4 ± 1.9	99.1 ± 2.0
* Limonin (ppm)	1.5 ± 0.1	<1 ± 0.1	<1 ± 0.1
Color	Golden	Golden	Pale golden
Flavour	Bitter orange marmalade	Bitter orange marmalade	Bitter orange marmalade
Aroma	Citrus	Citrus	Citrus
Cleanliness/defects	OK	OK	OK

(*) Tests carried out at 11.2 Brix degrees. Mean (*n* = 2) ± SD.

**Table 11:** Concentration of the main sugars present in the feed, permeate and reject/concentrate streams of the membranes studied.

Parameters	Feed Stream (COPE)	GR60PP Permeate	GR90PP Permeate	GR60PP Concentrate	GR90PP Concentrate
Brix degrees	22.0 ± 0.9	19.74 ± 0.79	18.40 ± 0.74	22.59 ± 0.90	22.24 ± 0.88
Fructose (g L^−1^)	55.63 ± 0.84	57.14 ± 0.85	52.85 ± 0.79	56.41 ± 0.85	57.04 ± 0.86
Glucose (g L^−1^)	61.99 ± 0.93	57.78 ± 0.87	55.64 ± 0.83	59.74 ± 0.89	60.90 ± 0.91
Sucrose (g L^−1^)	39.88 ± 0.60	36.40 ± 0.55	28.51 ± 0.43	42.48 ± 0.64	40.84 ± 0.61

**Table 12:** Concentration in sugars expressed at 11.2 Brix degrees of the feed, permeate and concentrate streams of the GR60PP and GR90PP membranes.

Sugars	Feed Stream (COPE)	GR60PP Permeate	GR90PP Permeate	GR60PP Concentrate	GR90PP Concentrate	Orange Juice Source: AIJN [75]
Fructose (g L^−1^)	28.32 ± 0.42	32.42 ± 0.48	32.17 ± 0.48	27.97 ± 0.42	28.73 ± 0.42	20–27 *
Glucose (g L^−1^)	31.56 ± 0.47	32.78 ± 0.49	33.87 ± 0.51	29.62 ± 0.44	30.67 ± 0.46	18–25 *
Sucrose (g L^−1^)	20.30 ± 0.30	20.65 ± 0.31	17.35 ± 0.26	21.06 ± 0.32	20.57 ± 0.31	*25–55*
Sucrose (%)	25.30 ± 2.11	24.06 ± 0.36	20.81 ± 0.31	26.78 ± 0.40	25.72 ± 0.39	*≤55*
Glucose/fructose	1.11 ± 0.02	1.01 ± 0.02	1.05 ± 0.02	1.06 ± 0.02	1.07 ± 0.02	*0.85–1.00*
Sugar-free extract	37.80 ± 0.57	32.55 ± 0.49	34.85 ± 0.52	39.63 ± 0.59	38.47 ± 0.58	*24–40*

(*) Mediterranean orange juices may contain glucose and fructose levels up to 35 g L^−1^. Mean (*n* = 2) ± SD.

**Table 13:** Results of sugar separation using membranes.

	GR60PP Permeate	GR90PP Permeate	GR60PP Concentrate	GR90PP Concentrate
	(%) Rejection		(%) Concentrate	
Fructose	−14.48 ± 0.22	−13.59 ± 0.20	1.24 ± 0.02	−1.43 ± 0.02
Glucose	−3.88 ± 0.06	−7.31 ± 0.11	6.15 ± 0.10	2.82 ± 0.04
Sucrose	−1.74 ± 0.02	14.51 ± 0.21	−3.75 ± 0.06	−1.31 ± 0.02

**Table 15:** Concentration of sugars present in the feed, permeate and concentrate stream.

Sample	Brix Degrees	Glucose g kg DM^−1^	Fructose g kg DM^−1^	Saccharose g kg DM^−1^
Feed stream (COPE)	18.99 ± 0.76	250.2 ± 3.75	249.2 ± 3.5	160.3 ± 1.2
Permeate stream	5.66 ± 0.22	359.8 ± 5.4	342.9 ± 5.8	-
Concentrate stream	39.78 ± 1.59	230.70 ± 3.46	224.50 ± 3.37	192 ± 3
Membrane rejection (%)	-	−43.8 ± 0.7	−37 ± 6	100
Concentration coefficient	-	1.44 ± 0.02	1.38 ± 0.02	-

## Data Availability

The original contributions presented in this study are included in the article/supplementary material. Further inquiries can be directed to the corresponding author(s).

## Supplementary Materials

The following supporting information can be downloaded at https://www.mdpi.com/article/10.3390/foods14244202/s1, Table S1: Evolution of control parameters during one of the tests carried out (UF module 100 kDa); Table S2: Operating conditions during the nanofiltration process.
