# Supplementary material for: Application of Membrane Technology to Obtain Bioactive Products from Orange Peel Extract
Source: Foods. 2025 Dec 7;14(24):4202. doi: 10.3390/foods14244202 (PMC12732071; doi:10.3390/foods14244202)
Supplement: Supplementary file 1 [file foods-14-04202-s001.zip › foods-4017272-supplementary.pdf]

Table S1. Evolution of control parameters during one of the tests carried out (UF module 100 kDa).

t (min)	Grados brix	Prod. (%)	T (°C)	P_1 (bar)	P_2 (bar)	ΔP (bar)	P_3 (bar)	Q_R (L/h)	Q_P (L/h)	Grados brix permeate
-	50.9	60	20	1	0	1	5	2900	-	-
					Dilutedwithwater					
-	40.2	30	30	1	0	1	2.5	3500	-	-
-	40.2	70	32	2.5	0	2.5	4	1150	-	-
					Dilutedwithwater					
-	30.7	70	38	2.4	0	2.4	3.5	2900	-	-
-	30.7	85	38	2.8	0.2	2.6	4.2	3200	-	-
					Dilutedwithwater					
0	24.3	60	38	2	0.2	1.8	3	3000	120	26.2
15	24.3	90	40	3.2	0.2	3	3.8	3500	150	26.38
30	24.3	90	44	3.2	0.2	3	3.8	3400	170	23.93
45	24.3	90	48	3.2	0.3	2.9	3.8	3400	**	22.86
60	24.6	90	50	3.2	0.4	2.8	3.8	3300	**	22.21
75	24.6	90	52	3.2	0.2	3	3.8	3300	150	22.13
90	24.6	90	56	3.2	0.2	3	3.8	3300	150	22.18
105	24.6	90	58	3.2	0.2	3	3.8	3300	**	22.13
					Raw material added					
120	26.5	90	50	3.2	0.2	3	3.8	3400	**	22.24
135	26.5	100	52	3.6	0.2	3.4	4.2	3500	**	23.05
150	26.5	100	54	3.6	0.2	3.4	4.2	3500	**	23.5
165	26.5	100	56	3.6	0.2	3.4	4.2	3400	**	23.48
180	26.5	100	58	3.6	0.2	3.4	4.2	3400	**	23.49
205	26.5	100	60	3.6	0.2	3.4	4.2	3400	**	23.61

Prod. (%).: *Proportion pumped to the membrane;* ***P_1:*** *Inlet pressure to the membrane;* ***P_2:*** *Outlet pressure from the membrane;* ***P_3:*** *Pump pressure at the membrane inlet;* ***ΔP:*** *difference between P_1 y P_2;* ***Q_R:*** *Recirculation flow rate;* ***Q_P:*** *Permeate flow rate.* *********The permeate flow meter did not register, but permeate flow was obtained.*

Table S2. Operating conditions during the nanofiltration process.

t (min)	Grados brix Concentrate	Grados brix Permeate	Temperature (°C)	P_1 (bar)	P_2 (bar)	ΔP (bar)	Q_R (L/h)	Q_P (L/h)	J_p(kg/m^2·h)
-	19.2	-	16.5	11.2	10.4	0.8	3500	-	-
-	19.6	-	16.8	20.3	19.6	0.7	3500	-	-
0	20.6	1.06	18.5	41.5	40.6	0.9	3500	170	68.09
15	25.3	1.54	25.7	39.4	39	0.4	3500	50	20.06
30	26.8	3.05	30.6	40.6	39.7	0.9	3500	36	14.53
45	28.1	4.35	34.6	39.9	39.2	0.7	3500	28	11.36
60	29.6	6.30	39.4	40.5	39.9	0.6	3500	27	11.04
75	30.7	8.22	43.2	39.4	38.8	0.6	3500	23	9.47
90	31.9	10.28	44.3	42.2	41.6	0.6	3500	22	9.14
105	32.9	10.89	45.3	40.9	40.3	0.6	3500	17	7.08
120	33.5	12.87	45.2	40.6	40	0.6	3500	3	1.26
135	34.6	13.80	44.6	41.2	40.4	0.6	3500	< 1	<1
150	36.7	14.39	46.6	50.1	49.4	0.7	3500	< 1	<1
175	38.6	14.33	50.3	50.2	49.6	0.6	3500	< 1	<1

P_1*:Inlet pressure to the membrane (bar);* ***P_2*:***Outlet pressure from the membrane (bar);* ***Q_R*:***Recirculation flow rate (L/h);* ***Q_P*:***Permeate flow rate (L/h);* ***J_p*:***Permeate mass flow rate (kg/h·m^2)*
